# The p68 and p72 DEAD box RNA helicases interact with HDAC1 and repress transcription in a promoter-specific manner

**DOI:** 10.1186/1471-2199-5-11

**Published:** 2004-08-06

**Authors:** Brian J Wilson, Gaynor J Bates, Samantha M Nicol, David J Gregory, Neil D Perkins, Frances V Fuller-Pace

**Affiliations:** 1 Department of Molecular and Cellular Pathology, Ninewells Medical School, University of Dundee, DD1 9SY, UK; 2 Division of Gene Regulation and Expression, School of Life Sciences, University of Dundee, Dow Street, DD1 5EH, UK; 3 Molecular Oncology Group – McGill University Health Centre, 687 Pine Avenue West, Montreal, Quebec, H3A 1A1, Canada; 4 Nuffield Department of Clinical Laboratory Sciences, John Radcliffe Hospital, University of Oxford, Oxford, OX3 9DU, UK; 5 Department of Microbiology and Immunology, McGill University, 3775 University Street, Montreal, H3A 2B4, Canada

## Abstract

**Background:**

p68 (Ddx5) and p72 (Ddx17) are highly related members of the DEAD box family and are established RNA helicases. They have been implicated in growth regulation and have been shown to be involved in both pre-mRNA and pre-rRNA processing. More recently, however, these proteins have been reported to act as transcriptional co-activators for estrogen-receptor alpha (ERα). Furthermore these proteins were shown to interact with co-activators p300/CBP and the RNA polymerase II holoenzyme. Taken together these reports suggest a role for p68 and p72 in transcriptional activation.

**Results:**

In this report we show that p68 and p72 can, in some contexts, act as transcriptional repressors. Targeting of p68 or p72 to constitutive promoters leads to repression of transcription; this repression is promoter-specific. Moreover both p68 and p72 associate with histone deacetylase 1 (HDAC1), a well-established transcriptional repression protein.

**Conclusions:**

It is therefore clear that p68 and p72 are important transcriptional regulators, functioning as co-activators and/or co-repressors depending on the context of the promoter and the transcriptional complex in which they exist.

## Background

The DEAD/H box family of RNA helicases has been demonstrated to be involved in virtually all processes that require manipulation of RNA including transcription, pre-mRNA and pre-rRNA processing, RNA export, ribosome assembly and translation [1]. Although, *in vitro*, several members of this family have been shown to unwind RNA duplexes, relatively few appear to be true processive helicases and it is clear that, in the cell, many are likely to be involved in unwinding of short base paired regions of RNA or in the modulation of RNA-protein interactions.

DNA helicases belong to a superfamily of proteins that is distantly related to DEAD/H box RNA helicases and includes the Werner syndrome protein (WRN) [2] and the Xeroderma pigmentosum XPB and XPD proteins [3], which have well established roles in transcription. Although the functions of DEAD/H box RNA helicases in other cellular processes, such as pre-mRNA processing and translation have been well studied, their role in transcriptional regulation is only now emerging. Examples of DEAD/H box RNA helicases involved in transcription include RNA helicase II (RHII/Gu) and RNA helicase A (RHA/NDHII). RHII/Gu was demonstrated to be a cofactor for c-Jun-activated transcription [4] and was shown to translocate from the nucleolus to the nucleoplasm after UV or anisomycin treatment (which activates JNK signalling). Although RHII/Gu was found to associate with phosphorylated c-Jun in a non-phosphorylated state, this association was observed to increase after anisomycin treatment, implying a stronger interaction when c-Jun is phosphorylated [4]. RHA is a homologue of the *Drosophila *maleless (MLE) gene product [5] and is thought to be important for gene dosage compensation on the X-chromosome [6]. RHA has been shown to be required for complex formation between the transcriptional co-activator, CREB binding protein (CBP), and RNA polymerase II [7]. Furthermore, different regions of the RNA helicase protein were found to interact with both CBP and RNA polymerase II. The association of RHA with RNA polymerase II was further investigated, and narrowed down to a 50 amino acid stretch, outwith the conserved helicase motifs [7]; this study also showed that RHA could regulate CREB-dependent transcription either through recruitment of Pol II or by ATP-dependent mechanisms. A later study reported that RHA acts as a bridging molecule between the breast tumour specific transcriptional activator, BRCA1 and the RNA polymerase II holoenzyme complex [8]. These reports thus provide clear evidence of a role for RNA helicases as transcription factors.

p68 is a prototypic member of the DEAD box family of proteins [9] and an established RNA helicase [10]. The subsequent discovery of p72 [11] and the finding that p68 and p72 share remarkable homology (90% over the central conserved core and 60% and 30% at the N- and C-terminal extensions respectively) suggests that these proteins may form a specific sub-group of DEAD box proteins and may have similar, but perhaps subtly different, functions in the cell, perhaps through interaction with different RNA substrates or proteins. *In vitro*, both proteins exhibit the RNA-dependent ATPase and RNA helicase activities characteristic of members of the DEAD box family [10-14] and have also been reported to catalyse rearrangement of RNA structure via branch migration [13]. Moreover p68 and p72 can interact with each other, as well as self-associate, and appear to preferentially form heterodimers in cells [15]. This provides the potential for a wide range of functions for p68 and p72 with the possibility of their co-operation in some contexts.

More recently p68 and p72 have been shown to be involved in a range of processes in the cell, including pre-mRNA and pre-rRNA processing and alternative splicing [16-18]. p68 and p72 have also been shown to be growth- and developmentally-regulated [19-22] and, furthermore, p68 appears to be over-expressed and poly-ubiquitylated in colorectal tumours [23]. Interestingly p68 has been shown to act as a transcriptional co-activator, specific for the activation function 1 (AF-1) domain of estrogen receptor alpha (ERα) [24]. This interaction was dependent upon phosphorylation of AF-1 at serine^118^, a residue phosphorylated by mitogen-activated protein kinase (MAPK). Interestingly the RNA helicase function of p68 appeared to be dispensable for this activity as a mutant p68 (Lys^144 ^to Arg) in the ATP binding site (conserved motif I) retained the ability to co-activate ERα, although in a later study RNA binding appeared to be required [25]. p72 was subsequently shown to share this property and both proteins were shown to interact with the activation domain 2 (AD2) of p160 co-activators [25]. Furthermore p68 was also found to interact with the CBP co-activator and RNA polymerase II [26]. More recently p68 has been shown to be recruited to the promoter of the ERα target gene pS2 [27], suggesting a direct involvement in transcriptional regulation.

In this report we explore further potential mechanisms through which p68 and p72 may contribute to transcriptional regulation. We find that, in some contexts, p68 and p72 can also act as transcriptional repressors and that these proteins exhibit clear promoter specificity in this function. By directing GAL4-tagged p68/p72 (GAL4 DNA binding domain aa1-147) to promoters containing GAL4 binding sites, we show that both p68 and p72 can repress transcription from the *herpes *virus thymidine kinase (TK) promoter but not the simian virus 40 promoter/enhancer. Moreover, while p72 can repress transcription from the *Adenovirus *major late promoter, p68 appears to have no effect, suggesting that these proteins do not behave in an identical way in all contexts. Furthermore we show, by co-immunoprecipitation, that both p68 and p72 interact with histone deacetylase 1 (HDAC1) and that HDAC1/p68/p72 co-elute by gel filtration, indicating that they exist in the same complex in the cell and suggesting a possible mechanism by which these proteins may exert their repressive effect.

## Results

### p68 and p72 differentially repress constitutively active promoters/enhancers

**Figure 1:**
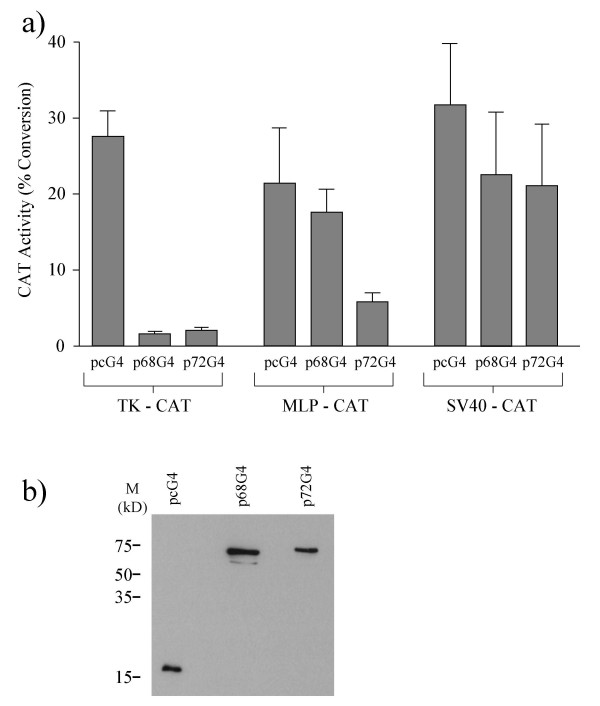
Effect of GAL4-tagged p68 and p72 on transcriptional activity as measured by CAT assays using the TK-CAT, MLP-CAT and SV40-CAT promoter-reporter plasmids, each harbouring 5 copies of a GAL4 binding site fused to the promoter. The pcDNA3-GAL4 expression vector (pcG4) was used as a control. In each case U2OS cells were co-transfected with pcG4 or plasmids expressing GAL4-tagged p68/p72 (p68G4/p72G4) and the appropriate promoter-reporter construct. The amounts of DNA transfected were: pcG4-, p68G4-/p72G4- 1 μg; TK-CAT- 2.5 μg; MLP-CAT- 9 μg; SV40-CAT- 0.5 μg. The amount of DNA used had been previously titrated to achieve appropriate levels of baseline CAT activity. a) Transcriptional repression by p68 and p72 as measured by CAT activity, which is shown as % conversion of ^14^C-labelled chloramphenicol to acetylated forms. The data represent results from 5 independent assays, which were each performed in triplicate. b) Western blot, using a GAL4-specific antibody, showing expression levels of the pcG4, p68G4 and p72G4 plasmids.

**Figure 2:**
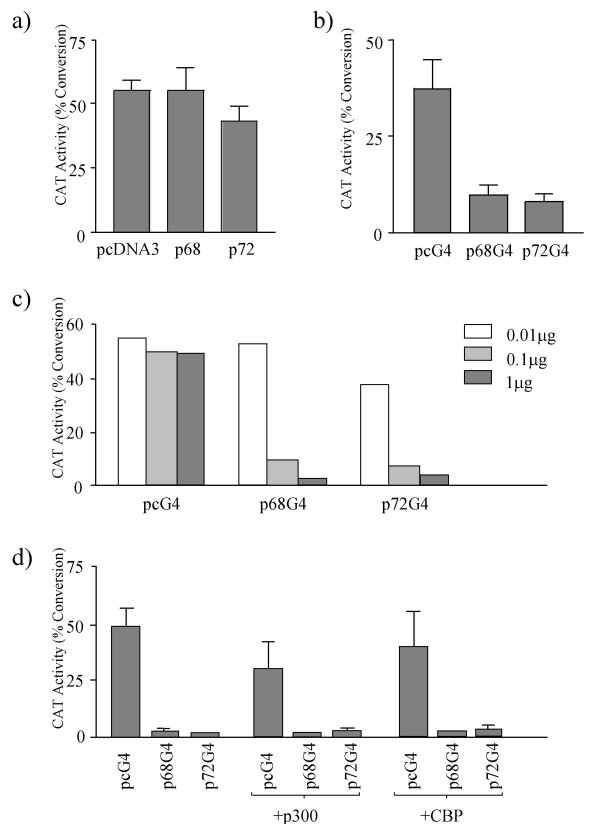
Control CAT assays to examine repression of the TK-CAT promoter-reporter in U2OS cells by p68/p72. The amounts of DNA transfected in each assay are indicated below and in all cases the % conversion of ^14^C-labelled chloramphenicol to acetylated forms is shown as an average of three independent experiments. a) Effect of untagged p68/p72 on TK-CAT transcription. 7.5 μg of control pcDNA3 vector, pcDNA3-p68 (p68) or pcDNA3-p72 (p72) were co-transfected with 2.5 μg of TK-CAT. b) Effect of GAL4-tagged p68 and p72 on transcriptional activity of a TK-CAT promoter-reporter which incorporated a 1.6 kb DNA 'spacer' between the GAL4 binding sites and the promoter (TK-S-CAT). 1 μg of pcDNA3-GAL4 (pcG4) or GAL4-tagged p68/p72 (p68G4/p72G4) were co-transfected with 5 μg of TK-S-CAT. The amount of TK-S-CAT had previously been titrated to achieve an appropriate baseline level of CAT activity. c) Titre of repression of TK-CAT activity by GAL4-tagged p68/p72. 2.5 μg of TK-CAT were co-transfected with different amounts of pcG4 vector, p68G4 and p72G4 as indicated. d) Effect of p300 and CBP on repression of TK-CAT transcription by GAL4-tagged p68/p72. 2.5 μg of TK-CAT were co-transfected with 1 μg of pcG4 vector, p68G4 or p72G4 together with 6.5 μg of either bluescript (as control) or p300/CBP.

In order to determine whether, in addition to their reported role as co-activator proteins [25], p68 and p72 helicases have any intrinsic transcriptional activity we generated plasmids to express p68-/p72-GAL4 DNA binding domain (aa1-147) fusion proteins (p68G4 and p72G4). U2OS cells were then co-transfected with p68G4, p72G4, or GAL4-tagged pcDNA3 (pcG4) plasmid as a control, and a *Herpes virus *thymidine kinase promoter/chloramphenicol acetyl transferase (CAT) reporter plasmid (TK-CAT) bearing 5 copies of the GAL4 binding site, and CAT activity was measured in standard assays. The amount of TK-CAT used had previously been titrated to give a basal level of activity within the linear range of measurement in the CAT assay system used (data not shown). Interestingly, we observed a marked decrease in CAT activity for both p68G4 and p72G4 (Figure 1a). This was confirmed using other cell lines, including MCF-7 and 293 HEK (data not shown). As many transcription factors have been shown to act differentially depending upon promoter context [28], we also tested the transcriptional activity of p68 and p72 with the *Adenovirus *major late promoter (MLP-CAT), and the SV40 promoter/enhancer (SV40-CAT). In each case we used the same amount of p68G4/p72G4 DNA as previously and appropriate amounts of the reporter constructs that would give similar basal levels of CAT activity using the GAL4 control plasmid (Figure 1a). The amounts of reporter plasmid DNA transfected had again been titrated previously to give similar basal levels, which were within the linear range (data not shown). We also confirmed that p68 and p72 were not limiting under these conditions (see Figure 2c and data not shown). Surprisingly p68 and p72 acted differentially with the MLP promoter, with p72 acting as a repressor, while p68 had no significant effect on CAT activity. Furthermore, neither p68 nor p72 repressed transcription from the simian virus 40 promoter/enhancer (SV40-CAT-Figure 1a). Western blotting, using an antibody against GAL4 confirmed that p68 and p72 were expressed at similar levels in these cells (Figure 1b). Taken together these results reveal not only that p68 and p72 appear to have a previously unknown transcriptional repressive ability, but also that this activity is variable depending on the promoter context.

### The repression of transcription is an active process

To confirm that the repressive effect observed is not due to an artefact of the assay conditions we initially repeated the experiment with the TK-CAT reporter plasmid, using non-tagged p68 or p72 or the pcDNA3 expression plasmid vector alone (Figure 2a). In this experiment neither p68 nor p72 significantly reduce transcription of the TK-CAT reporter, suggesting that the repression observed with the GAL4-tagged p68/p72 plasmids (Figure 1) is not due to competing out of an essential factor required for TK-CAT transcription but, instead, implies an active mechanism of repression in which the p68/p72 proteins are required to be directed to the TK-CAT promoter via the GAL4 tag.

The possibility still remained that p68 and p72 were repressing transcription by direct interference with the transcriptional machinery of the TK-CAT promoter (e.g. perhaps by physically blocking the promoter). To rule out this possibility, we used a similar TK-CAT promoter construct, which had, however, a 1.6 kb DNA 'spacer' between the GAL4 binding sites and the promoter (the inserted DNA is reported to have no effect on transcription [29]). Both GAL4-p68 and -p72 retained the ability to repress transcription, almost to the same degree as previously (Figure 2b). Furthermore, a titration using the GAL4- p68/p72 fusion proteins clearly shows a dose-dependent concentration curve (Figure 2c) as would be expected for active repression. In addition, p68 and p72 have been observed to interact with p300/CBP co-activators [24,26]. Therefore it remained possible that the observed transcriptional repression was due to competition for, or interference with, p300/CBP. If this were the case co-expression of p300/CBP would be expected to relieve repression by p68/p72. As shown in Figure 2d, no such relief of expression was observed. Thus our findings that transcriptional repression by p68/p72 was dose-dependent and not due to steric blocking of the promoter or competition/interference with p300/CBP, suggest that it is an active process.

### Deletion experiments do not identify a distinct repression domain for p68 or p72 but reveal an activation domain

**Figure 3:**
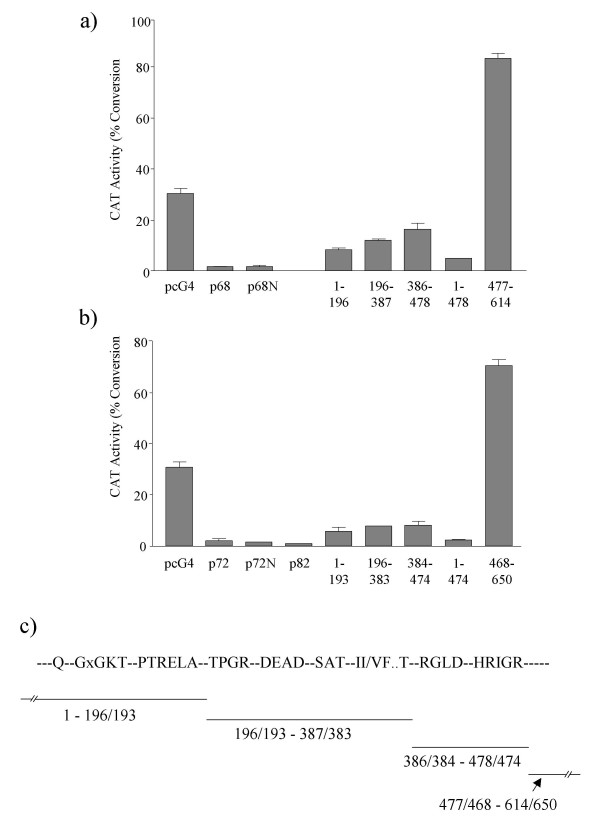
Deletion mapping of potential repression/activation domains in a) p68 and b) p72 as observed in CAT assays using the TK-CAT promoter-reporter plasmid. The pcDNA3-GAL4 expression vector (pcG4) and full-length GAL4-tagged p68/p72 were used as controls. All p68/72 deletion derivatives were expressed as GAL4-tagged fusion proteins in pcG4 and included the amino acids indicated. Additional proteins tested in this assay included the ATPase/helicase GAL4-tagged inactive mutants of p68/p72 (p68N/p72N) and the alternative upstream initiation product of the p72 gene (p82). The amounts of DNA used in the transfections were; TK-CAT- 2.5 μg; pcG4 and all p68/p72 constructs- 1 μg. The % conversion of ^14^C-labelled chloramphenicol to acetylated forms is shown as an average of five independent experiments. c) Diagram correlating the deletion end-points to the position of the motifs conserved in the DEAD box family of proteins.

To determine whether specific regions or domains of p68 and p72 act as transcriptional repressors, we performed CAT assays, again using the TK-CAT reporter, but with a range of deletion derivatives of p68/p72 covering the entire coding region. Deletion derivatives encompassing domains between amino acids 1–478 and 1–474 for p68 and p72 respectively repressed transcription in this assay while the C-terminal region for both proteins (aa 477–614 for p68 and 468–650 for p72) acted as a strong transcriptional activator (Figure 3a,3b). Residues 1–478 of p68 and 1–474 of p72 include all the conserved motifs that characterise the DEAD box family of proteins (Figure 3c). Within this conserved core we have shown that there are three domains, which can independently repress transcription (Figure 3c), while the complete region (aa 1–478/474) can repress as well as the full-length respective proteins. Additionally, the finding that the C-terminal regions of p68 and p72 activate transcription is consistent with earlier reports of transcriptional activation by p68/p72 [25,26]. Interestingly, ATPase inactive mutants of p68 and p72 (in which the DEAD motif had been mutated to NEAD) as well as the more recently identified p82 (a derivative of p72 which uses an alternative non-AUG upstream translation initiation codon [30]) also repress transcription (Figure 3b). Thus ATPase and helicase activity appear to be dispensable for transcriptional repression suggesting that this function of p68/p72 may not specifically require RNA unwinding; again this is consistent with reports that helicase activity is not required for co-activation of ERα transcriptional activity [24,25] although another report suggested that p68 helicase activity is required for synergism with the transcriptional co-activators CBP/p300 [26].

### p72 immunoprecipitates a HDAC activity

**Figure 4:**
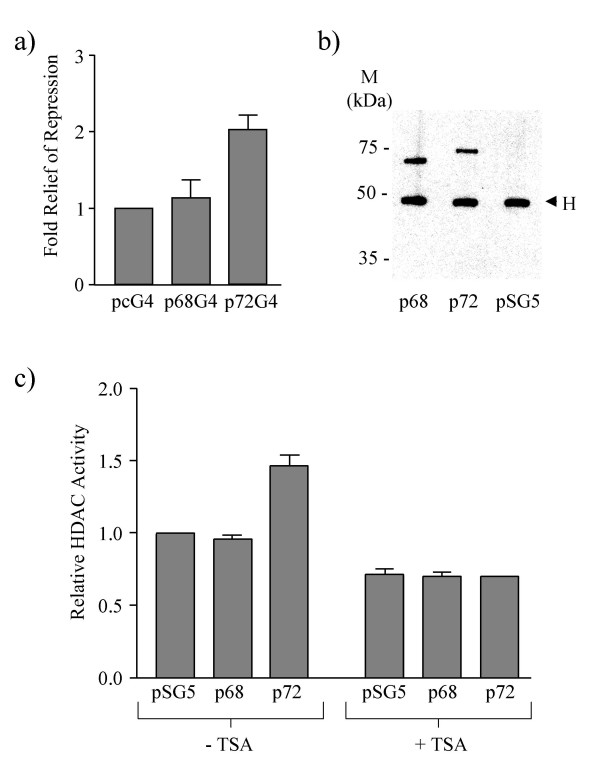
The involvement of HDAC activity in transcriptional repression by p68/p72. a) Relief of p68/p72 repression of MLP-CAT transcription by TSA. 1 μg of pcDNA3-GAL4 (pcG4) or GAL4-tagged p68/p72 (p68G4/p72G4) were co-transfected with 9 μg of MLP-CAT and TSA was added 16 hr after transfection, at a final concentration of 300 nM. The values for p68G4 and p72G4 are given relative to the baseline value for the pcG4 vector control, which was set at 1, and represent the average from three experiments. b) Immunoprecipitation/western blotting of myc-tagged p68 and p72 from 293 cells expressing these proteins. Myc-tagged proteins were immunoprecipitated with the anti-myc epitope antibody, 9E10, and western blotted with the same antibody to detect the presence of p68-myc and p72-myc fusion proteins. A myc-tagged pSG5 vector control is included. pSG5, p68 and p72 all refer to myc-tagged versions. H denotes cross reaction with the antibody heavy chain. Molecular weight markers (in kDa) are indicated. Equal amounts of these immunoprecipitated proteins were used in the HDAC activity assay shown in c. c) HDAC activity assay of immunoprecipitated p68 and p72 (see b). HDAC activity in the presence and absence of TSA is shown relative to that of the myc-tagged pSG5 vector control, which was set at 1, and represent the average from three experiments.

Many studies have implicated histone deacetlyase (HDAC) proteins in active repression of transcription, and many transcriptional repressors have been shown to associate with HDACs [31]. To test whether the observed transcriptional repression by p68 and p72 was dependent on HDAC activity, CAT activity assays were performed as before, using the TK-CAT and MLP-CAT reporter plasmids, in the presence and absence of the HDAC inhibitor trichostatin A (TSA). The relief of repression by TSA was then determined for p68-/p72-GAL4 compared with that observed for the GAL4 vector alone since TSA will also increase basal levels of transcription. No effect was observed with the TK-CAT promoter (data not shown). For the MLP-CAT promoter repression was relieved two-fold in the case of p72 while no effect was seen with p68 (Figure 4a). This is not surprising since p68 does not repress transcription from this promoter (Figure 1). These findings therefore suggest that HDAC activity appears to be important for transcriptional repression of the MLP promoter by p72 and imply that repression of the TK-CAT may employ a different mechanism [32].

To examine further the involvement of HDACs in transcriptional repression by p68/p72 we determined whether p68 and/or p72 co-immunoprecipitate a HDAC activity in cells. Plasmids expressing myc-tagged p68 and p72 were expressed in 293 cells (with myc tag plasmid vector as a negative control) and the tagged proteins were immunoprecipitated from cell lysates using the myc epitope antibody, 9E10. Equal amounts of immunoprecipitated protein bound to the antibody, as confirmed by western blotting (Figure 4b), were then used in an HDAC activity assay (Biomol) in the presence and absence of TSA. In this assay (Figure 4c) p72 was found to co-immunoprecipitate a HDAC activity, which is abolished by TSA, while p68 did not. These findings are consistent with p72 interacting with a HDAC and repressing transcription in a HDAC-dependent manner.

### p68 and p72 associate with HDAC1 in cells

**Figure 5:**
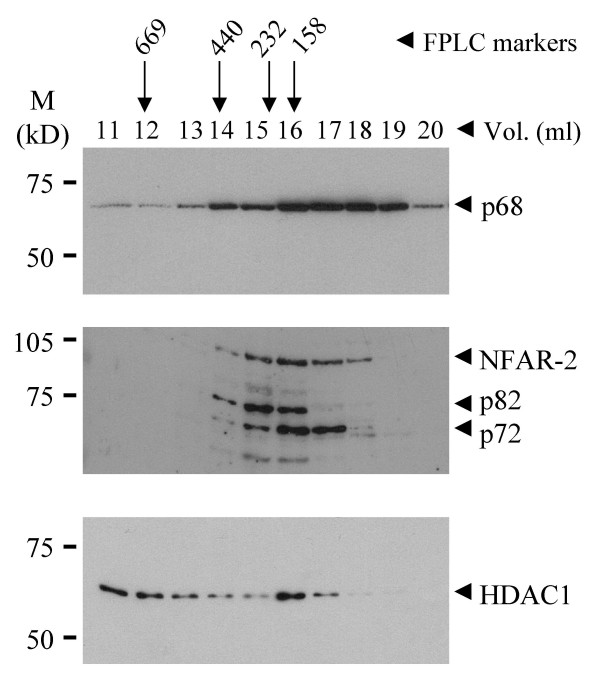
Western blots showing gel filtration elution profiles of p68, p72 and HDAC1. p68, p72 and HDAC1 in the fractions were detected by western blotting using appropriate antibodies. Note that the antibody raised against p72 also recognises p82 and cross-reacts with NFAR-2 [15]. All lysates had been treated with DNase and RNase prior to gel filtration. The void volume and elution position of the Pharmacia FPLC size markers are indicated, as are molecular weight markers (in kDa).

**Figure 6:**
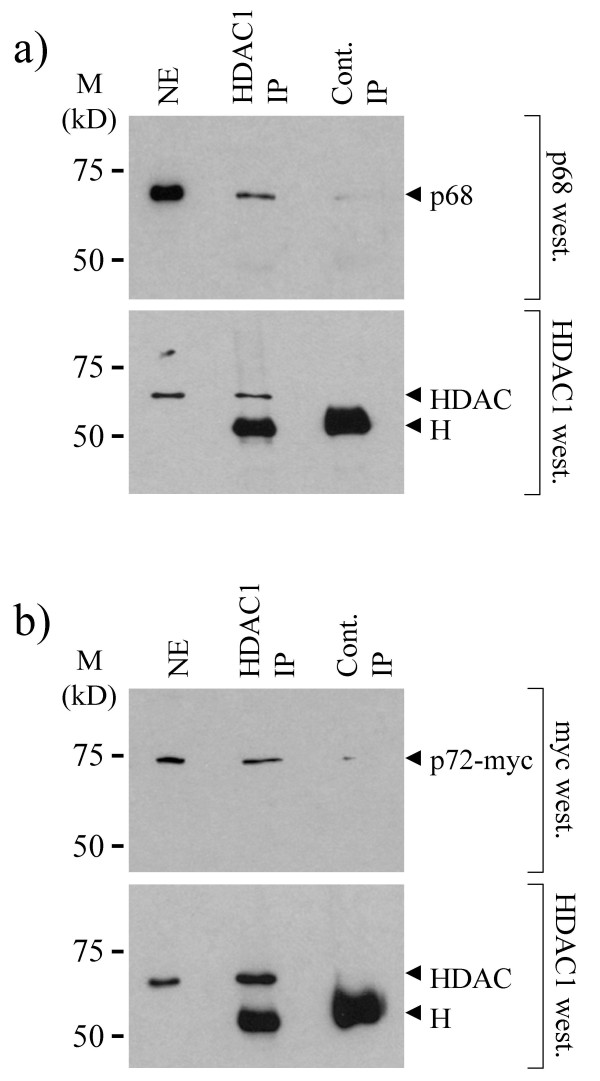
Co-immunoprecipitation of a) p68 and b) p72 with HDAC1. a) HDAC1 was immunoprecipitated from U2OS nuclear extracts using an HDAC1-specific antibody. Immunoprecipitated proteins were separated by SDS-PAGE and the presence of HDAC1 and associated p68 was detected by western blotting with p68- and HDAC1-specific antibodies. b) HDAC1 was immunoprecipitated from nuclear extracts of 293 cells expressing myc-tagged p72 using an HDAC1-specific antibody. Immunoprecipitated proteins were separated by SDS-PAGE and the presence of HDAC1 and associated myc-tagged p72 was detected by western blotting with HDAC1- and myc epitope- specific antibodies. In both experiments a control immunoprecipitation (IP) was performed using an irrelevant rabbit IgG. An aliquot of nuclear extract (NE) was also included in the western blots (west.) as an additional control. H denotes cross reaction with the antibody heavy chain. Molecular weight markers (in kDa) are indicated.

**Figure 7:**
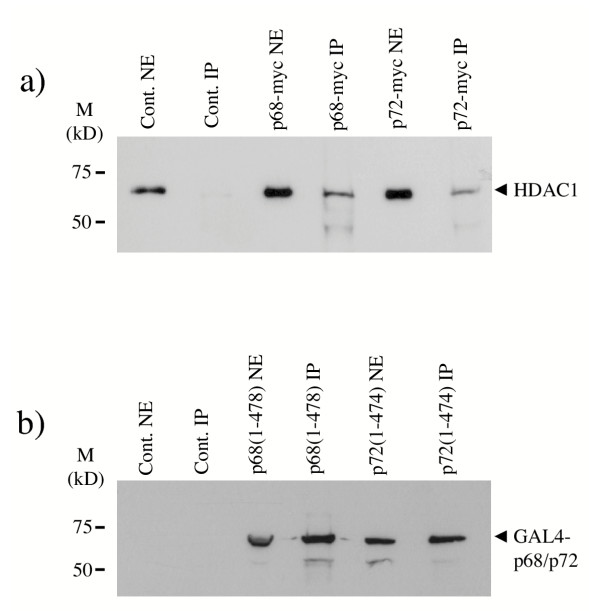
a) Reciprocal co-immunoprecipitation of p68 and p72 with HDAC1. Myc-tagged p68 and p72 were immunoprecipitated from nuclear extracts of 293 cells expressing these proteins using a myc epitope-specific antibody and associated HDAC1 was detected by western blotting with an HDAC1-specific antibody. 293 cells, which had not been transfected, were used as control. b) Co-immunopreciptiation of p68/p72 deletion derivatives with HDAC1. HDAC1 was immunoprecipitated from nuclear extracts of 293 cells expressing GAL4-tagged p68 and p72 deletion derivatives, which encompass residues 1–478 and 1–474 of p68 and p72 respectively. Associated p68/p72 were detected by western blotting with a GAL4-specific antibody. 293 cells, which had not been transfected, were used as control. NE-nuclear extract, IP-immunopreciptiation. Molecular weight markers (in kDa) are indicated.

Three classes of HDACs have been described. Class I HDACs, which include HDAC1, 2 and 8, are expressed in the nucleus and have been shown to bind several transcription factors and to mediate transcriptional repression [31]. Since HDAC1 is the prototypical member (in mammalian cells) and has been well studied we decided to examine whether p68 and/or p72 associate with HDAC1 in cells. We had previously shown that a large proportion of p68 and p72 co-elute by gel filtration [15]. Therefore we examined fractions from a DNAse/RNAse-treated 293 cell lysate, which had been separated by gel filtration, by western blotting using antibodies against p68, p72 and HDAC1, and showed that a significant proportion of HDAC1 co-elutes with the majority of p72 and a substantial proportion of p68 in the cell in complexes that are of a size consistent with p68/p72 interacting with HDAC1 (Figure 5). Since co-elution does not, in itself, indicate an interaction, we went on to examine whether p68 and or p72 co-immunoprecipitate with HDAC1 from cell lysates. For p68, nuclear extracts were prepared from U2OS cells and HDAC1 was immunoprecipitated with an HDAC1-specific antibody. Immunopreciptiated proteins were then separated by SDS-PAGE and the presence of p68 was detected by western blotting with a p68-specific antibody (Figure 6a). As currently available antibodies against p72 cross react with other nuclear proteins [15] 293 cells were transfected with myc-tagged p72 and interactions between the myc-tagged p72 and HDAC1 were examined. In this case, therefore, nuclear extracts were prepared from transfected cells, HDAC1 was immunoprecipitated as before and associated myc-tagged p72 was detected by western blotting using the myc epitope-specific antibody, 9E10 (Figure 6b). In each case, the presence of HDAC1 in the immunoprecipitate was confirmed by western blotting with the HDAC1-specific antibody (Figure 6a,6b). As an additional control we carried out a reciprocal co-immunoprecipitation experiment in which nuclear extracts from 293 cells transfected with myc-tagged p68/p72 were prepared, the myc-tagged p68/p72 proteins were immunoprecipitated using the myc epitope-specific antibody and associated HDAC1 was detected by western blotting using the HDAC1-specific antibody (Figure 7a). Cells that had not been transfected were used as a control. Additionally, since deletion derivatives encompassing residues 1–478 of p68 and 1–474 of p72 can repress transcription as well as the respective full-length proteins, we examined whether these deletions could co-immunoprecipitate with HDAC1. We therefore transfected 293 cells with GAL4-tagged proteins containing residues 1–478 of p68 and 1–474 of p72, prepared nuclear extracts, immunoprecipitated HDAC1 and western blotted for associated GAL4-tagged p68/p72 with a GAL4-specific antibody. As shown in Figure 7b, these deletion derivatives co-immunoprecipitate efficiently with HDAC1, suggesting that this region of p68/72 interacts with HDAC1. These findings thus indicate that p68 and p72 associate with HDAC1 in cells and that the interaction appears to be mediated by the regions which, in our system, are responsible for the transcriptional repression activity of p68 and p72. Moreover, since the immunoprecipitations were performed with extracts that had been treated with DNase/RNase (see Materials and Methods), the interactions of p68/p72 and HDAC1 are not mediated by nucleic acid.

## Discussion

We have shown that the highly related DEAD box RNA helicases p68 and p72 act as repressors of transcription in a promoter-context manner. When targeted to the TK-CAT promoter-reporter construct they both strongly repress transcription (Figure 1). Furthermore, this transcriptional repression does not appear to be due to either squelching or physical blocking of the transcription apparatus (Figure 2), implying an active transcriptional mechanism. Moreover repression of TK-CAT was observed in several cell lines (U2OS, 293, MCF-7) suggesting that it is not cell line dependent. In order to determine whether this repression activity exhibited any promoter specificity we also tested the ability of p68 and p72 to repress transcription of other constitutively active promoter-reporter constructs with high basal levels of transcription, namely MLP-CAT and SV40-CAT. Interestingly MLP-CAT revealed a difference in the ability of p68 and p72 to repress transcription, with p72 strongly repressing transcription of this promoter-reporter and p68 having no effect (Figure 1). This observation suggests that, although highly homologous (70% overall identity at the amino acid level [11]) p68 and p72 proteins may act differently in some contexts, perhaps through the association with different protein partners. Neither p68 nor p72 repressed transcription of SV40-CAT (Figure 1) suggesting that the repression by p68 and p72 is promoter context-dependent, an observation that has been reported for other transcription factors [33]. These findings thus are consistent with the observed repression activity of p68/p72 being an active process. Interestingly, in this context, another DEAD box protein DP103 (Ddx20) has been found to act as a co-repressor of the Ets repressor METS/PE1 [34].

Using a series of deletion derivatives of p68 and p72 we identified three domains, within the core conserved among the DEAD box family of proteins, which can independently repress transcription (Figure 3c). Moreover regions encompassing residues 1–478 of p68 and 1–474 of p72, which contain the complete conserved core (Figure 3c), can repress transcription as well as the respective full-length proteins (Figure 3a,3b). In contrast, the C-terminal extension of both proteins acts as a transcriptional activator in this context (Figure 3a,5) consistent with earlier reports of these proteins acting as transcriptional co-activators [24,25]. Thus, using this system, we have shown that there are separable transcriptional repression and activation domains within p68 and p72.

Since HDAC proteins have been extensively implicated in the repression of transcription, it was important to examine whether these proteins are likely to play a role in transcriptional repression by p68 and/or p72. Firstly, the ability of the HDAC inhibitor, TSA, to relieve repression was examined. No effect was observed on repression of TK-CAT (data not shown) implying the involvement of a HDAC-independent mechanism in repression of the TK promoter. In contrast, repression of MLP-CAT by p72 was relieved two-fold compared with the vector control (Figure 4a) suggesting the involvement of HDACs in this process. (Since p68 did not repress MLP-CAT transcription, the lack of effect by TSA is not surprising.) Supporting these data, p72 was found to co-immunoprecipitate a HDAC activity which was abolished by the addition of TSA, while p68 did not (Figure 4b). We chose to investigate whether p68 and/or p72 associate with HDAC1, since it is a well-studied example of Class I HDACs. Both p68 and p72 co-immunoprecipitate with HDAC1 (Figures 6 and 7); furthermore HDAC1, p68 and p72 co-elute in similar sized complexes by gel-filtration, which are of an appropriate size (Figure 5) supporting the idea of interactions between p68/p72 and HDAC1 in cells. Moreover, the finding that these proteins co-immunoprecipitate and co-elute from extracts which had been treated with DNase/RNase suggest that these represent protein-protein interactions rather than merely interactions via nucleic acid. While an interaction between HDAC1 and p68 is not supported by the results of the HDAC assay, or the TSA experiment, it is possible that, in some instances, p68 does recruit HDAC1 and that this mechanism is not being triggered in the MLP-CAT assay or HDAC assay. Alternatively, it is possible that the observed co-immunoprecipitation of p68 and HDAC1 is occurring through the interaction between p68 and p72 [15] or that HDAC1 associated with p68 may have other, possibly non-transcriptional, roles [35,36]. However, the data are entirely consistent with p72 recruiting HDAC1 to achieve active repression of transcription. Future investigations should also reveal whether the differential ability of p68 and p72 to recruit *active *HDAC proteins is responsible for the difference observed upon the MLP promoter.

Our attempts at correlating the different repressive functions of p68 and p72 to specific domains of the respective proteins, using deletion derivatives, were unsuccessful, as the equivalent regions of both either caused transcriptional repression or activation. While this might suggest that both helicases repress transcription in the same manner, it is more likely that the HDAC recruitment by p72 may be an additional mechanism of repression, used at specific promoters. We also cannot rule out the recruitment of other repression complexes at this stage. Our findings that p68 and p72 differ in their ability to repress the MLP promoter and to recruit HDAC activity suggest that, at least in some contexts, p68 and p72 repress transcription by different mechanisms.

In summary, it is clear that p68 and p72 act to repress transcription in a differential manner dependent upon promoter context. It will be important to determine which endogenous promoters are subject to repression by p68/p72 in a physiological context. However, until the signal transduction pathways, which target these proteins to the appropriate promoters, are elucidated it will be necessary to use a targeting system (such as GAL4) to undertake a molecular analysis of transcriptional repression by p68 and p72. Since it is now clear that p68/p72 can act both to activate and repress transcription future work will involve dissection of the transcriptional activation/repression complexes in which p68 and p72 are involved, as well as characterisation of the molecular 'switch' which determines whether these proteins will be part of transcriptional activation or repression complexes.

## Conclusions

We have shown that the highly related RNA helicases p68 and p72 can repress transcription in a promoter context-dependent manner. Both proteins associate with HDAC1, a well-established transcriptional repressor protein. Strikingly, however, p68 and p72 behave differently in their ability to repress transcription from different promoters and in their ability to recruit HDAC activity suggesting that they may, at least in some contexts, repress transcription by different mechanisms.

## Methods

### Cell culture

U2OS human osteosarcoma cells and 293 human embryo kidney cells were maintained at 5% CO_2 _at 37°C in DMEM with 10% FBS, 2 mM L-glutamine, 100 units/ml penicillin and 100 μg/ml streptomycin (all supplied by Invitrogen).

### Plasmids

A pcDNA3-GAL4 expression plasmid was used to express full-length and deletion derivatives of p68/p72/p82 tagged at the N-terminus with the DNA binding domain (amino acids 1–147) of GAL4 [37]. The majority of deletion derivatives were created by PCR and inserted, as *Bam*HI/*Eco*RI fragments, in frame with the GAL4 tag. The CAT reporter constructs have been described previously: TK-CAT [38], TK-Spacer-CAT [29], MLP-CAT and SV40-CAT [39]. The MLP-CAT and SV40-CAT plasmids were a kind gift from Douglas Dean (Washington University, St Louis, USA), and the TK-spacer-CAT plasmid was kindly provided by Dr. Alain Nepveu (McGill University, Canada). Untagged p300, CBP, p68, p72 were expressed from pcDNA3. A myc-tagged derivative of pSG5 (Stratagene) [15] was used to express the myc-tagged p68/p72 or the myc epitope alone as negative control.

### Antibodies

p68: The antibodies used were the mouse monoclonal antibody PAb 204 and the rabbit polyclonal antibody 2906, generated against the C-terminal 15 amino acids of p68 [19]. PAb 204 was originally generated against the SV40 large T antigen but it cross-reacts with p68 [9]. It is specific for p68 in cells that are not infected or transformed by SV40. p72: A rabbit anti-peptide polyclonal antibody generated against amino acids 624 to 638 [15] was used to detect p72/p82 in fractions from gel filtration. Myc epitope: A mouse monoclonal antibody (9E10) was used to detect proteins tagged with the myc epitope (MRQKLISEEDL). HDAC1: A rabbit polyclonal antibody (Oncogene Research Products) was used both for immunoprecipitation and western blotting. GAL4- a mouse monoclonal antibody (Santa Cruz) was used to detect GAL4-tagged proteins. A negative control rabbit IgG antibody for immunoprecipitation was obtained from R&D systems and appropriate anti-mouse and anti-rabbit secondary antibodies were obtained from DAKO.

### Transient transfections and chloramphenicol acetyl-transferase (CAT) assays

3×10^5 ^U2OS cells were seeded for transfections, which were performed as previously described [38]. Cells were co-transfected with the appropriate CAT reporter construct and GAL4-tagged p68/p72, and in each case the DNA was made up to a total of 10 μg with Bluescript DNA (Stratagene). CAT activity was determined 48 hr after transfection using 100 μg of total protein from cleared whole cell lysates. Typically each experiment was performed in triplicate within each assay, and each assay was repeated 3 to 5 times. Where applicable, the HDAC inhibitor Trichostatin A (TSA) (Upstate Biotechnology) was added, at a final concentration of 300 nM, 16 hr post transfection.

### Nuclear extract preparation and co-immunoprecipitation

Nuclear extracts were prepared from U2OS and 293 cells essentially as described in [40] except that the NaCl concentration was reduced to 330 mM NaCl, then diluted to 150 mM NaCl (after nuclear lysis) and treated with RNAse/DNAse. The extract was pre-cleared in buffer D [20 mM Hepes (pH7.9), 150 mM NaCl, 0.5 mM DTT, 20% (v/v) glycerol, 10 mM NaF and protease inhibitor cocktail (Roche)] with protein G sepharose beads for 30 min. at 4°C. Immunoprecipitations were carried out in the presence of appropriate antibodies and protein G sepharose beads for one hour at 4°C. After washing in buffer D plus 0.1% Igepal (Sigma), immunoprecipitated proteins were separated by SDS-PAGE and western blotted using standard conditions and appropriate primary and secondary antibodies. Immunoreactive proteins were detected using the ECL method (Amersham).

### Gel filtration

293 cell lysates were prepared in RIPA buffer [50 mM Tris-HCl (pH8.0), 150 mM NaCl, 1% Igepal, 0.1% SDS, 1% Na deoxycholate, 1 mM EDTA and protease inhibitor cocktail (Roche)]. After treatment with RNase/DNase, lysates were fractionated on a Pharmacia Superose 6 HR column in 50 mM Tris-HCl (pH7.5), 150 mM NaCl, 10% glycerol and 1 mM benzamidine, using a Pharmacia AKTA FPLC system. 0.5 ml fractions were collected and alternate fractions were analysed by western blotting. Molecular weight standards from Pharmacia were used to calibrate the column.

### HDAC assay

293 cells were transfected with either pSG5-myc vector, pSG5-p68-myc, or pSG5-p72-myc. Cells were lysed in buffer B 48 hr after transfection. The lysate was then diluted in buffer A and myc-tagged proteins immunoprecipitated with 9E10 (myc epitope) antibody as described above. The HDAC assay employs *Fleur de lys *substrate, which contains an acetylated lysine side chain (Biomol) and was performed according to manufacturers instructions. Antibody-bound beads were washed in HDAC assay buffer prior to being added to the 96-well plate, to remove immunoprecipitation buffer. Reactions were incubated for 30 min at 37°C with or without the addition of 1 μM TSA. Samples were excited at 360 nm and emitted at 460 nm and were read in a fluorometer. Typically each assay was performed 3 times.

## Authors' contributions

BJW carried out the transcriptional and HDAC activity assays, the deletion mapping and the gel filtration, GJB and SMN carried out the co-immunoprecipitation experiments, DJG carried out the original experiments showing that p68 and p72 could act as transcriptional repressors, and NDP and FFP participated in the design of this study. FFP co-ordinated the study and prepared the final draft of the manuscript. All authors read and approved the manuscript.
